# Use of the right to information act to improve access to better services in two urban slums

**DOI:** 10.1186/1753-6561-6-S1-O6

**Published:** 2012-01-16

**Authors:** Rajarajeshwari, DV Sowmya Bharadwaj, KV Ramamurthy

**Affiliations:** 1 CIVIC, Bangalore 560 025, India

## Introduction

In the context of inflationary trends and the incapacity of the poor to access services in open markets, the Indian government initiated various programmes to provide basic facilities either free or at nominal cost. However, these services are not reaching the most vulnerable people due to a lack of awareness, barriers in accessibility and rampant corruption in the system.

The failure in basic service delivery has impacted the health and economy of the poor, especially in urban areas such as Bangalore. The urban poor are unable to access food grains in the open market, which prevents them from attaining the minimum required nutrition, and makes them vulnerable to health problems.

A similar situation exists in health services. It is necessary to build pressure to improve accessibility for public services - especially public distribution system, primary health centres and *Anganwadis* (supplementary nutrition centres) to improve health in urban areas. In Bangalore, though there are various service points to provide services on health, the quality of the service delivery at the service points are not assessed. Most of the time, these service points don't work as per norms such as working hours, availability of allocated supplies to that service point, and appropriate service delivery methods, leading to a failure of the realisation of the whole entitlement reserved for the needy.

## Methods

As part of our work in urban governance reforms, we undertook a rights-based approach in two selected slums of Bangalore to address a few major services that were provided to the poor under subsidy or for free, namely, food and health.

We documented the experience with using the right to information act (RTI) as a tool to push governance reforms in specific departments and initiate dialogue with service agencies to develop charters and enhance delivery of services through improving/developing platforms such as grievance redressal meetings.

## Results

We found that using RTI as a tool helped with the establishment of *anganwadi* centres (supplementary nutrition centres) in two slums. There was an improvement of food provision in the existing centres for children, pregnant and lactating women. The coverage of schemes like the newly introduced *Bhagya Lakshmi* scheme (financial assurance scheme for girl child) increased.

The regularity of visits by the auxiliary nurse midwife and lady health visitor from the health sub-centre also improved. The coordination between the *Anganwadi* centre and health sub-centre also improved.

In the case of the public distribution system (PDS), the confidence in the community to demand their entitlements under the scheme increased. This also improved the attitude of the PDS shop owners towards the community especially with respect to their willingness to increase the frequency and working hours of shop. Beneficiaries reported an increase in the quantity of food grain distributed in accordance with the rules, thus decreasing the burden. Families that were eligible to receive below poverty level cards have applied for these cards.

## Discussion

To bring changes in delivery of basic services such as health and PDS, a multifaceted approach needs to be adopted with awareness on entitlements as a basis. We faced many challenges in obtaining cooperation from the service agencies in accessing information on the details of entitlements of the poor for specific service points.

RTI is a key tool, through which it was possible to bring high level of change in the two urban slums. Other tools such as citizens' charters, grievance redressal mechanisms, and government orders were also needed.

